# Comparative N-Glycoproteomic and Phosphoproteomic Profiling of Human Placental Plasma Membrane between Normal and Preeclampsia Pregnancies with High-Resolution Mass Spectrometry

**DOI:** 10.1371/journal.pone.0080480

**Published:** 2013-11-15

**Authors:** Fuqiang Wang, Ling Wang, Zhonghua Shi, Gaolin Liang

**Affiliations:** 1 CAS Key Laboratory of Soft Matter Chemistry, Department of Chemistry, University of Science and Technology of China, Anhui, China; 2 State Key Laboratory of Reproductive Medicine, Analysis Center, Nanjing Medical University, Nanjing, Jiangsu, China; University of Tennessee Health Science Center, United States of America

## Abstract

Preeclampsia is a serious complication of pregnancy, which affects 2–8% of all pregnancies and is one of the leading causes of maternal and perinatal mortality and morbidity worldwide. To better understand the molecular mechanisms involved in pathological development of placenta in preeclampsia, we used high-resolution LC-MS/MS technologies to construct a comparative N-glycoproteomic and phosphoproteomic profiling of human placental plasma membrane in normal and preeclamptic pregnancies. A total of 1027 N-glyco- and 2094 phospho- sites were detected in human placental plasma membrane, and 5 N-glyco- and 38 phospho- proteins, respectively, with differentially expression were definitively identified between control and preeclamptic placental plasma membrane. Further bioinformatics analysis indicated that these differentially expressed proteins correlate with several specific cellular processes occurring during pathological changes of preeclamptic placental plasma membrane.

## Introduction

The mechanisms involved in maintaining a human pregnancy, or the switches between the normal pregnancy outcome and adverse outcome such as miscarriage, preeclampsia, fetal growth restriction, or preterm labor, are complicated. But the role of the placenta in these processes is very crucial [1], [2]. During a healthy pregnancy, maternal spiral arteries are dramatically remodeled. They become widely dilated and lose their responsiveness to vasoconstrictive stimuli. Thus blood enters the intervillous space in a non-pulsatile manner and even under a low blood pressure [1], [2]. Preeclampsia (PE) affects about 2 to 3% of all pregnancies but this number can be even higher in underdeveloped countries. It is an important cause of maternal death worldwide and a leading cause of iatrogenic prematurity and fetal growth restriction. In PE, spiral artery remodeling is partial or incomplete [3], [4]. The ensuing high pressure flow results in hydrostatic damage to the placental villi. Furthermore perfusion by intermittent pulses of fully oxygenated arterial blood is thought to lead to fluctuations in oxygen delivery and subsequently result in oxidative stress [4]. The maternal syndrome is, at least in part, caused by the maternal response to this placenta damage. This is known as the two-stage model of preeclampsia [4], [5].

Plasma membrane (PM) is a selective permeable barrier and communication interface of cells owing to the presence of specific membrane proteins, which play important biological and pharmacological roles in intercellular communication, cellular development, cell migration, cell recognition, immune response, and other functions [5], [6]. Therefore, study on PM is helpful to understand the functions of normal or PE placentas. Glycoproteins are proteins that contain oligosaccharide chains (glycans) covalently attaching to the polypeptide side-chains. Phosphoproteins are proteins that are physically bonded to a substance containing phosphoric acid [6], [7], [8]. Both glycosylation and phosphorylation of a protein happen during its cotranslational or post-translational modification and latter controls protein folding, conformational distribution, stability, and activity. Additionally, most proteins secreted into plasma from disease tissue are glycosylated or phosphorylated, and thus they could be important disease biomarkers (e.g., preeclampsia). Moreover, glycoproteins and phosphoproteins are often important integral PM proteins, playing important roles in a lot of key processes such as cell–cell interactions, molecule recognition, etc [9], [10], [11]. Therefore in this work, we conducted the glycosylation and phosphorylation analyses of these important proteins.

Although several proteomic studies on the human placenta have been reported [12], [13], [14], [15], to the best of our knowledge, the N-glycoproteomic and phosphoproteomic profiling of human placenta plasma membrane has not been reported. In this work, we aim to establish strategies for the identifications of N-glyco- and phospho- proteins including quantifications of N-glyco- and phospho- proteins, and characterizations of the glycan- and phospho- sites of glycoproteins and phosphoproteins in placental PM of normal and PE pregnancies. Further bioinformatics analyses of these proteins will provide researchers with deeper insights into some molecular and cellular processes during the pathological changes of placenta PM in preeclampsia.

## Materials and Methods

### Sample Preparation

All samples and clinical information were collected at the Nanjing Maternity and Child Health Care Hospital of Nanjing Medical University. All patients provided written informed consent. This study was approved by the Ethics Committee of Nanjing Medical University with an Institutional Review Board (IRB) number of 2012-NFLZ-32. PE was defined as a systolic blood pressure of (or above) 150 mmHg or diastolic blood pressure of (or above) 110 mmHg on two occasions in six hours. The detailed patient characteristics are presented in supplemental Table S1. For each placenta sample, 0.5 g of tissue was dissected from the maternal side of the placentas (in the central part, exclusive of calcified area) and rinsed in 0.9% saline, then frozen in liquid nitrogen prior to use. 20 normal placentas and 20 placentas of pregnant women with pre-eclampsia were collected. Placentas from the normal and pre-eclampsia women were randomly divided into three groups, 6, 7, 7 of each, respectively. Then the 6 groups of samples were pooled separately and subjected to phosphorylation and glycosylation proteomics analysis. The phosphorylation and glycosylation proteomics analysis was repeated three times.

### Plasma Membrane Preparation

In total, 10 g isolated placenta tissue was homogenized in 20 mL cold Plasma membrane isolation buffer consisting of 1 mM CaCl_2_, 50 mM HEPES, 0.1 mM phenylmthylsulfonyl fluoride, 1 mM EDTA, and 1% (w/v) protease inhibitor cocktail as stock solution. For each sampling, 1 mL of homogenate consisting of approximate 1 g tissue was added with 4 mL Plasma membrane isolation buffer for further homogenization. The pH value was adjusted to 7.4 with KOH. The homogenate was centrifuged at 600×g for 10 min at 4 °C. The supernatant was separated and centrifuged at 24000×g (Beckman, SW28 rotor) for another 30 min at 4 °C. The supernatant was discarded, and then the pellets were transferred to sw-28 tubes and then added with 50% sucrose until a final concentration of 42.3% (1.45 M). A two-layer step gradient (0.25 M/1.35 M sucrose) was prepared to enrich plasma membrane suspension. After being applied on the gradient and centrifuged at 100000g (Beckman, SW28 rotor) for 2.5 h, the plasma membrane was located at the interface of the 0.25 M/1.35 M sucrose gradient and appeared as one band. Then the sample band was collected for subsequent analysis [16], [17].

### Glycoprotein Digestion, Dimethyl Labelling, Glycopeptide Enrichment and Release of Glycans by PNGase F

The pure PM pellet was solubilized with 2% SDS, 8 M urea in 25 mM NH_4_HCO_3_ (pH 8.0). One milligram of placenta PM protein from normal or Preeclampsia placenta was reduced with 10 mM DTT at 60°C for 1 h, and alkylated with 55 mM IAA at 37 °C for 40 min. The tryptic peptides were desalted with homemade C18 solid phase extraction column and then dried down in a speed Vac (eppendof, USA), then resuspended in 100 µL of triethylammonium bicarbonate (100 mM). Subsequently, formaldehyde-H2 (573 µmol) was added to above solution and vortexed for 2 min followed by the addition of freshly prepared sodium cyanoborohydride (278 µmol). The resultant mixture was vortexed for 60 min at room temperature (RT) and then a total of 60 µL of ammonia (25%) was added to consume the excess formaldehyde. Finally, 50 µL formic acid (50%, Sigma) was added to acidify the solution. For heavy labeling, ^13^C-D_2_-formaldehyde (573 µmol) and freshly prepared cyanoborodeuteride (278 µmol) were used. The light and heavy dimethyl-labeled samples were mixed at 1∶1 ratio based on total peptide amount, which was determined by running an aliquot of the labeled samples on a regular LC-MS/MS run and comparing overall peptide signal intensities. The dried labeled peptides were reconstituted in CWR buffer (8 uL Concanavalin A (conA, Canavalia ensiformis), 15 uL wheat germ agglutinin (WGA, Triticum vulgaris), 9 uL Ricinus communis Agglutinin 120 (RCA120)), centrifuged at 500 rpm for 1 min, then kept in the dark for 1 h at 4°C. The mixture was then centrifuged and filtered with molecular weight cut off (MWCO) of 1000 Dalton at 14000×g for 25 min. After that, 200 uL BB (1 mM MnCl_2_ in 40 mM Tris/HCl, pH 7.6) was added and the mixture was centrifuged at 14000×g and 4 °C for 25 min. This procedure was repeated for 3 times. Then the supernatant was discarded and the pellet was added with 50 uL of 50 mM Ammonium bicarbonate (ABC), centrifuged at 14000×g and 4°C for 215 min, repeated twice. The glycans were released from glycopeptides after the addition of 500 units of PNGase F (New England biolabs, USA) in 40 uL ABC to the resin and being incubated at 37 °C for 3 hours with gentle shaking. The deglycosylated peptides were carefully collected after gentle centrifugation.

### Acquisition of Mass Spectrometry Data and Glycopeptide Identification

The labeled deglycosylated peptides were applied on the LTQ-Orbitrap instrument (Thermo Fisher, USA) equipped with a Waters Nano ACQUITY UPLC system via a nanospray source for data acquisition. The LC-MS/MS was operated in positive ion mode. The analytical condition was set at a linear gradient from 0 to 60% of buffer B (CH_3_CN) in 150 min, and flow rate of 200 nL/min. For analysis of PM from human placeta, one full MS scan was followed by five MS/MS scans on those five highest peaks respectively. The MS/MS spectra acquired from precursor ions were submitted to Maxquant (version 1.2.2.5) using the following search parameters: the database searched was Uniprot proteome (version20120418); the enzyme was trypsin (KR/P); the dynamic modifications were set for oxidized Met (+16) and Deamidation ^18^O (Asn); carbamidomethylation of cysteine was set as static modification; MS/MS tolerance was set at 10ppm; the minimum peptide length was 6; the false detection rate for peptides, proteins, and Deamidation ^18^O were all set below 0.01.

### Phosphoprotein Digestion, Dimethyl Labelling, and Phosphopeptides Enrichment

The pure PM pellete was solubilized with 100 uL radioimmunoprecipitation assay (RIPA) lysis buffer containing 1 uL 100× stcok solution of protease inhibitor cocktail, 20 mM NaF, 20 mM Na_3_V_4_. Procedures for the digestion of phosphoproteins and dimethyl labeling were same as those described above. The labeled peptides were then diluted with 6% TFA in 50% (v/v) CH_3_CN prior to enrichment experiment; 0.1 mg Ti-doped mesoporous silica (Ti-MPS) was dispersed into 200 uL labeled phosphoprotein digests and shaked for 30 min at room temperature. The phosphopeptide-binding Ti-MPS materials were recovered by centrifugation and rinsed with 100 uL solution of 0.1% TFA in 80% (v/v) CH_3_CN twice to remove nonspecifically adsorbed peptides. The bound phosphopeptides were released from the beads by sonication of the beads in 100 uL 5% NH_3_•H_2_O for 10 min.

### Acquisition of Mass Spectrometry Data and Phosphopeptide Identification

The MS/MS spectra acquisition of the labeled phosphopeptides was same as that of glycopeptides described above. The MS/MS spectra acquired from precursor ions were submitted to Maxquant (version 1.2.2.5) using the following search parameters: the database searched was Uniprot proteome (version20120418); the enzyme was trypsin (KR/P); the dynamic modifications were set for oxidized Met (+16), Deamidation ^18^O (Asn); phosphorylated Ser, Thr, and Try (+80); carbamidomethylation of cysteine was set as static modification; MS/MS tolerance was set at 10 ppm; the minimum peptide length was 6; the false detection rate for peptides, proteins, and phosphorylation were all set below 0.01.

### Bioinformatics analysis

To further explore the significance of the N-glyco- and phospho- proteins, we used Pathway Studio (v5.00) software (Ariadne Genomics,MD, USA) to search the relevant molecular functions and cellular processes of these identified proteins during the pathological changes of placenta PM. KEGG pathway and GO analysis were also performed for the N-glyco- and phospho- proteins. We used following equations to calculate the enrichment ratio and *p*-value for GO analysis and define N as total number of genes annotated by GO in whole genome, n as total number of genes annotated by a specific GO term in whole genome, M as total number of genes annotated by GO in N-glyco- and phospho- proteins, and m as total number of genes annotated by a specific GO term in N-glyco- and phospho- proteins.
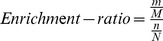


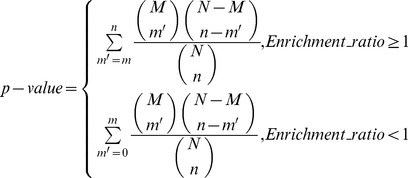



### Immunohistochemical Analyses

For immunolocalization of ENG (Abcam ab70993, Cambridge, UK; 1:200), placenta sections (5 µm thickness) were obtained and fixed with 4% (w/v) paraformaldehyde (or 10% (w/v) formaldehyde) in PBS for 10 min. Then the slides were washed with PBS, blocked in 5% BSA solution for 30 min at 37 °C and incubated with primary antibodies overnight at 4 °C. Excess antibodies were removed by incubation of the slides with 0.1% Tween-20 in TBS for 15 min. Then the sections were incubated with biotinylated and streptomycin-labeled goat anti-mouse or rabbit antibody (Maixin Bio, KIT-5010, Fujian, China) for 15 minutes at room temperature. After 3 washes with TBST, the expression of the proteins in placenta sections was detected by the reaction of the second antibody with peroxidase and 3,3,9 -diaminobenzidine etrahydrochloride (DAB), and then analyzed under an Olympus BX61 fluorescence microscope.

## Results

### Detection of the Pathological Changes of Placenta PM in Preeclamptic Pregnancies

**Figure 1:**
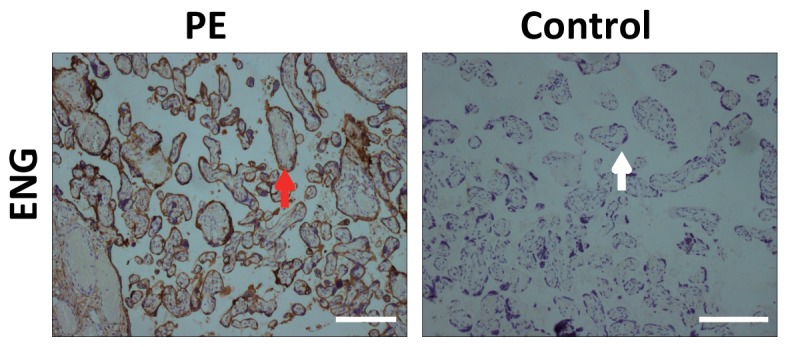
Immunohistochemical analyses of ENG expression in the placentas from preeclamptic and normal pregnancies. ENG protein was localized in the syncytiotrophoblast layer of both control (right, white arrow) and preeclamptic placentas (left, red arrow). ENG staining is increased in placentas from preeclampsia. Scale bar: 100 µm.

We confirmed that the representative pathological changes of placenta PM between normal and preeclamptic pregnancies were consistent with those previously reported [18], [19]. As shown in Figure 1, ENG protein was localized in the syncytiotrophoblast layer of both control (right, white arrow) and preeclamptic placentas (left, red arrow) while the PE placentas had obviously increased expression of ENG compared with that in normal placentas. All the placenta samples from PE and control pregnancies were identified by ENG immunohistochemical analyses before N-glyco- and phospho- proteomic analyses.

### Global N-glycoproteomic and Phosphoproteomic Profiling of Human Placenta Plasma Membrane

**Figure 2:**
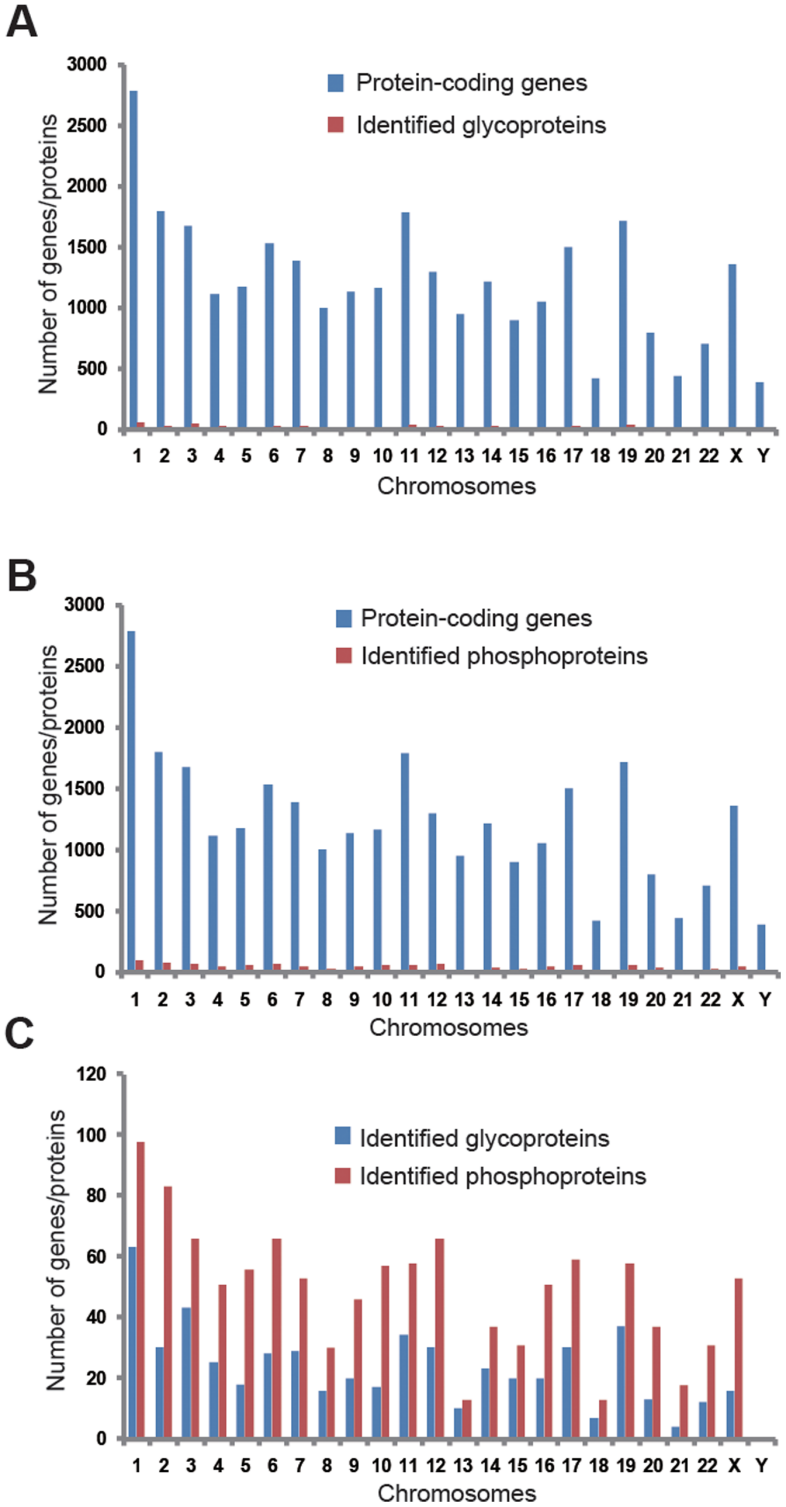
Comparison of the number of protein-coding genes in chromosomes with those of identified glycoproteins (A) and phosphoproteins (B) in chromosomes with LC-MS/MS in this work. Comparison of the number of identified glycoproteins with that of phosphoproteins (C) in chromosomes.

Protein glycosylation and phosphorylation are major post-translational modifications that control protein folding, conformational distribution, stability, and activity [5], [6], [7]. Even though several proteomic studies on the human placenta have been reported, to the best of our knowledge, the N-glycoproteomic and phosphoproteomic profiling of human placenta plasma membrane has not been reported [11], [13], [14], [15]. Thus, herein we intend to establish strategies for N-glyco- and phospho- protein identification in human placenta PM. By the large-scale proteomic analysis, we identified 1027 N-glyco- and 2094 phospho- sites in the human placental PM with high confidence (two or more unique peptides with an FDR less than 1%, Table S2, Figure S1 and Figure S2). The distribution of these proteins in different chromosomes were analyzed and compared to those of protein-coding genes in human chromosomes. As shown in Figure 2A and 2B, the distributions of these identified proteins are totally different from that of human protein-coding genes in chromosomes (Table S3). Interestingly, we found that these identified N-glyco- and phospho- proteins have similar distribution patterns in human chromosomes (Figure 2C).

**Figure 3:**
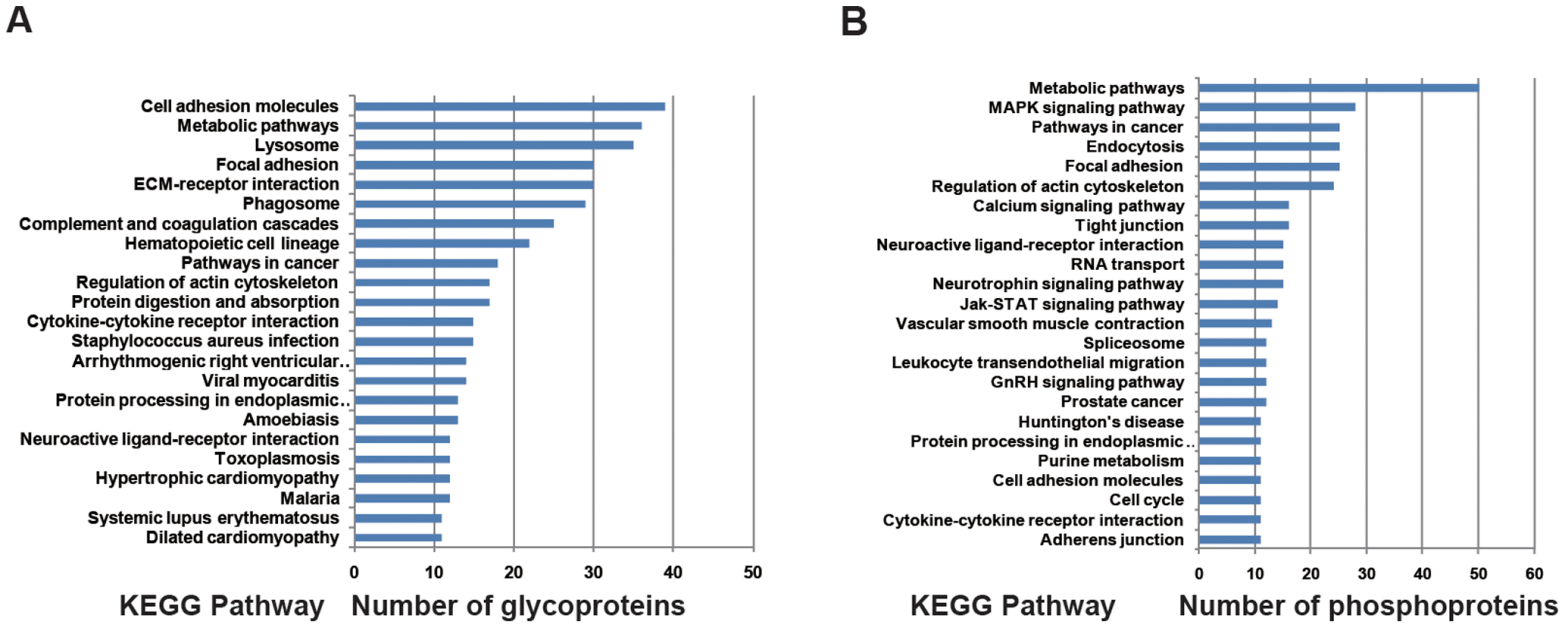
Representative significant biological pathways in which detected placental proteins are predicted to be involved. KEGG pathway analysis was performed using the identified placental placental plasma glycoproteins (A) and phosphoproteins (B) to evaluate which pathways are significantly represented (*p* < 0.05 and counts of the linked proteins > 10).

KEGG pathway analyses were performed on the identified proteins to identify the significantly represented pathways in the human placental PM that they are involved in. 23 and 24 pathways were identified for these N-glyco- and phospho- proteins respectively, as shown in Figure 3A and 3B (*P* < 0.05 and counts of the linked proteins > 10). Most of these identified pathways are related with the functions of placental PM during pregnancy, according to previous research. For example, 39 glycosylated proteins are predicted to be related with the cell adhesion pathway (Figure 3A) and 50 phosphorylated proteins are predicted to be related with the metabolic pathway (Figure 3B). Since placenta PM is the critical channel between the mother and fetus for oxygen and nutrients transportation, the facts that these proteins were associated with cell adhesion pathway and metabolic pathway could be well anticipated.

### Identification of N-glyco- and Phospho- Proteins Related to Pathological Development of Placenta PM in Patients with PE

To detect the differentially expressed N-glyco- and phospho- proteins between the placenta samples from normal and preeclamptic pregnancies, we examined the expression patterns of the 1027 N-glyco- and 2049 phospho- sites identified respectively. Analysis of the mass spectrometry data from LTQ-Orbitarp-Velos with Maxquant (version 1.2.2.5) revealed that there are 7 N-glyco protein peptides and 42 phospho- protein peptides differentially expressed in normal and PE placenta PM. After date analysis, we found that these proteins peptides correspond to 5 N-glyco- (Table 1) and 38 phospho- proteins (Table 2) differentially expressed, respectively.

**Table 1:** The list of differentailly expressed N-glyco- proteins in human placenta plasma membrane from control and preeclamptic pregnancies.

Protein name	*p*-value	Fold change
Hemopexin	0.01	–3.53
Fibromodulin	0.042	–1.78
Stromal interaction molecule 1	0.029	2.01
EMILIN-1	0.014	2.38
Rho GTPase-activating protein 11A	0.004	32.62

**Table 2:** The list of differentailly expressed phospho- proteins in human placenta plasma membrane from control and preeclamptic pregnancies.

Protein name	*p*-value	Fold change
Phosphoglucomutase-like protein 5	0.007	–11.89
Filamin-A	0.000	–9.61
Cysteine and glycine-rich protein 1	0.000	–8.6
Talin-1	0.008	–5.27
Ras-related protein Rab-39B	0.045	–4.38
Hemoglobin subunit gamma-1	0.004	–4.37
KN motif and ankyrin repeat domain-containing protein 2	0.002	–4.2
Hemoglobin subunit gamma-1	0.000	–3.68
Hemoglobin subunit gamma-2	0.001	–3.39
Vinculin	0.002	–2.85
Myosin light chain kinase,smooth muscle	0.003	–2.53
Kininogen-1	0.000	–1.88
Cyclin-Y	0.001	–1.86
Calnexin	0.022	–1.58
Coiled-coil domain-containing protein 6	0.033	–1.39
Nuclear mitotic apparatus protein 1	0.036	1.44
Serine/arginine-rich splicing factor 2	0.000	1.44
G antigen family C member 1	0.044	1.59
TH domain-containing protein 1	0.000	1.61
Hepatoma-derived growth factor	0.035	1.64
CapZ-interacting protein	0.001	1.7
Ubiquitin carboxyl-terminal hydrolase 24	0.036	1.8
Bcl-2-associated transcription factor 1	0.006	1.81
Cordon-bleu protein-like 1	0.009	1.82
Tetratricopeptide repeat protein 21A	0.004	1.83
Thyroid hormone receptor-associated protein	0.000	1.97
40S ribosomal protein S3	0.001	2.01
Programmed cell death protein 4	0.001	2.03
Transforming acidic coiled-coil-containing protein 2	0.036	2.44
Serine/arginine repetitive matrix protein2	0.009	2.93
Prelamin-A/C	0.014	3.1
Serine/arginine repetitive matrix protein1	0.005	3.18
Serine/arginine repetitive matrix protein1	0.006	3.19
Neuroblast differentiation-associated protein AHNAK	0.019	3.22
Prelamin-A/C	0.000	3.43
Prelamin-A/C	0.000	3.99
Isoform 2 of Srcsubstrate cortactin	0.019	3.99
Bcl-2-associated transcription factor 1	0.026	5.05

### Bioinformatics Analysis of Differentially Expressed N-glyco- and Phospho- Proteins

After further characterization the specific and unique expression patterns of these 5 N-glyco- and 38 phospho- proteins, we subsequently grouped these proteins into two clusters according to the expression patterns (increased expression pattern and decreased expression pattern). Among these 5 N-glyco-proteins, 2 proteins have increased expression patterns while the other 3 proteins have decreased expression patterns. In the mean time, 15 phospho- proteins have increased expression patterns in the PE placenta PM, while the expression patterns of the other 23 proteins decreased. After that, these identified 5 N-glyco- and 38 phospho- proteins were subjected to KEGG Pathway (Table 3) and GO analysis (Table 4) for further identification of important biological processes that they were significantly involved in. Indeed we found these biological processes are all present in PE development. The predicted KEGG pathways were ranked according to their *p*-values and we found that the most significant biological processes include Focal adhesion, Shigellosis, and Bacterial invasion of epithelial cells (Table 3). For GO analysis, the top ten representative biological processes were listed in Table 4. To further analyze the the networks between important cellular processes and these differentially expressed N-glyco- and phospho- proteins, we used Pathway StudioTM for pathway analysis. The results indicate that these proteins are involved in pathways of “Fetal Death ", “Essential hypertension ", and “Hypertension”.

**Table 3:** Representative significant biological pathways in which detected placental N-glyco- and phospho- proteins are predicted to be involved.

Pathway name	Counts	Fold	*p*-value
Focal adhesion	4	9.53	0.002
Shigellosis	2	15.54	0.009
Bacterial invasion of epithelial cells	2	13.54	0.012

**Table 4:** Go analysis for the differentially expressed N-glyco- and phospho- proteins.

Representative biological processes	Enrichment fold	*p*-value
Negative regulation of DNA repair	489.4	0.002
Sterol regulatory element binding protein import into nucleus	489.4	0.002
Positive regulation of response to interferon-gamma	489.4	0.002
Actin crosslink formation	244.7	0.004
Aorta smooth muscle tissue morphogenesis	244.7	0.004
Positive regulation of cell aging	244.7	0.004
Transforming growth factor beta receptor complex assembly	163.1	0.006
Establishment or maintenance of microtubule cytoskeleton polarity	163.1	0.006
Activation of store-operated calcium channel activity	163.1	0.006
Heme metabolic process	163.1	0.006

## Discussion

Preeclampsia is a specific disorder known to promote maternal or perinatal mortality and morbidity during pregnancy [2], [3], [4], [5], [6]. Several vast bodies of literature have suggested that preeclampsia could be associated with many factors such as endothelial dysfunction, inflammation, and insulin resistance, although its etiology and pathogenesis have not been fully elucidated [3], [4] [6].

Several stable isotope labeling-based quantification methods have been used in combination with N-glycoproteomic and phosphoproteomics approaches, including chemical labeling such as iTRAQ [20], [21], [22], and metabolic labeling such as SILAC [23], [24]. The advantage of a chemical modification approach over metabolic labeling is its ability to chemically label tissue samples after the samples are lysed and digested. This makes the approach more generically applicable since it also allows the quantitative analysis of biological samples that cannot be easily cultured, such as human placental. Furthermore, quantification at the peptide level allows separated analyses of glycosylation and phosphorylation events on the same protein. Here, we introduced stable isotope dimethyl labeling for the quantitative analyses of N-glyco- and phospho peptides. In addition, stable isotope dimethyl labeling costs less, but has comparable outcomes [25]. None the less, mass spectrometry has limitations regarding quantification of protein amounts. Multiple operational procedures inherent to the assay (e.g., protein extraction, digestion, labeling, enrichment, etc) negatively affect its reproducibility. Moreover, the relatively low resolution of MS limits accurate detection of proteins that are not at least moderately aboundant. Considering these limitations of the technique, we obtained the mass data from three independent and consistent experiments to increase the reliability.

In this work, we performed comparative proteome studies to determine the N-glyco- and phospho- proteins differentially expressed in human placenta between normal and preeclamptic pregnancies. In total, 1027 N-glyco- and 2094 phospho- sites were detected in human placenta PM, among which 7 protein peptides having difference in glycosylation and 42 protein peptides having difference in phosphorylation between two groups were successfully identified which correspond to 5 N-glyco- and 38 phospho- proteins, respectively. Further expression pattern analysis indicated that there were two distinct expression patterns for the above mentioned proteins (i.e., increased expression pattern and decreased expression pattern). Bioinformatics analysis provided abundant functional information of the identified proteins. Identification of these differentially expressed proteins in placenta PM of PE patients during the pathological development provides a useful resource for deep study of the biological factors that influence the pathological development of placenta.

Here listed are those pathways involving the glycosylated and phosphorylated proteins indentified (Figure 3) and those relating to the pathological development of placental PM in PE (Table 3). First, metabolic pathways are differentiated not only by virtue of different enzyme systems that catalyze their forth and back reactions (anabolism and catabolism), but also by multiple compartmentalization [26], [27]. Aqueous compartments such as cytoplasm, extracellular fluid, the mitochondrial matrix, or the endoplasmatic reticulum lumen are separated from each others by membranes or phospholipid bilayers. Weakly hydrophobic molecules (e.g. molecular oxygen O_2_, carbon dioxide CO_2_, steroid hormones, and thyroxin) rapidly diffuse across these membranes via free diffusion [28], [29]. Water has a slow rate of diffusion and its diffusion is facilitated by the presence of aquaporins (water channels) [30]. The first stage of PE is characterized with placental hypoxia due to a relative reduction in uteroplacental blood flow, which is resulted from restricted trophoblast invasion [31]. In reverse, hypoxia is also an essential element for the success of invasion. Under hypoxic conditions, 2-methoxyestradiol (2-ME) could induce cytotrophoblast cells to differentiate into an invasive phenotype [30], [32]. 2-ME is generated by catechol-O-methyltransferase, an enzyme involved in the metabolic pathway of estrogens. During pregnancy, circulating 2-ME levels increase significantly compared to those in menstrual cycle. Interestingly, plasma levels of 2-ME are lower in women with PE than in controls, and these differences become obvious weeks or even months before the clinical manifestations of PE [31], [32], [33]. Second, Focal adhesions (also cell–matrix adhesions or FAs) are specific types of large macromolecular assemblies through which both mechanical force and regulatory signals are transmitted [34], [35]. More precisely, they can be considered as the processes that sub-cellular macromolecules mediate the regulatory effects (e.g. cell anchorage) of extracellular matrix (ECM) adhesion [36]. Focal adhesions serve as the mechanical linkages to the ECM, and as a biochemical signaling hub to concentrate and direct numerous signaling proteins at the sites of integrin binding and clustering. They are limited within the clearly defined ranges of a cell, from plasma membrane to 15 nm of the ECM substrate surrounded. Trophoblast differentiation during the first trimester of pregnancy includes cell proliferation and invasion, and extracellular matrix (ECM) remodeling [37]. Researches have indicated that, in a variety of cell types, processes such as proliferation, invasion, and ECM remodeling require the turnover of focal adhesions mediated by a cytoplasmic tyrosine kinase named focal adhesion kinase (FAK).

In the list of differentially expressed N-glyco- and phospho- proteins (Table 1 and 2), several proteins play key roles in the incidence and development of PE such as PAGE4 and EMILIN1. PAGE4 is a member of the GAGE family. The GAGE genes are expressed in a variety of tumors and in some fetal and reproductive tissues. They are overexpressed in prostate cancer, testicular cancer, and uterine cancer. They are also expressed in some male and female reproductive tissues including prostate, testis, fallopian tube, uterus, and placenta [38]. PAGE4 is a highly (100%) intrinsically disordered protein (IDP). Its primary protein sequence conforms to the features of a typical IDP sequence and its secondary structure prediction with algorithm metaPrDOS strongly supports this hypothesis. Furthermore, overexpressed PAGE4 protects cells from stress-induced death [38], [39], [40]. The EMILIN1 genes encode an extracellular matrix glycoprotein characterized by an N-terminal microfibril interface domain, a coiled-coiled alpha-helical domain, a collagenous domain, and a C-terminal globular C1q domain [41]. This protein associates with elastic fibers at the interface between elastin and microfibrils and plays an important role in the development of elastic tissues including large blood vessels, dermis, heart, and lung. The detection of EMILIN1, at the level of the ectoplacental cone and trophoblast giant cells of developing mouse embryos, suggested that this protein plays a structural and functional role in the process of placentation [41]. During the establishment of human placenta, a highly migratory subpopulation of extravillous trophoblasts (EVT), originating from anchoring chorionic villi, penetrate and invade the uterine wall. EMILIN1, produced by decidual stromal and smooth muscle uterine cells, is expressed in the stroma. In some instances, it has an increasing gradient of concentration in the perivascular region of modified vessels. This distribution pattern is consistent with the haptotactic directional migration observed in in vitro functional studies of freshly isolated EVT and of the immortalized HTR-8/SVneo cell line of trophoblasts [41], [42]. Function-blocking using monoclonal antibodies against alpha4-integrin chain and against EMILIN1 or EMILIN1-specific short interfering RNA confirmed that trophoblasts interact with EMILIN1 or its functional gC1q1 domain via α_4_β_1_ integrin [42], [43]. Finally, membrane type I-matrix metalloproteinase (MT1-MMP) and MMP-2 were upregulated in co-cultures of trophoblast cells and stromal cells, suggesting that EMILIN1 contributes to the haptotactic process [41], [42], [43].

In summary, through comparative proteome analysis of human placenta plasma membrane from normal and preeclamptic pregnancies, we constructed the N-glyco- and phospho- protein expression profiles. We identified markers of important cellular processes, and suggest several factors that may be involved in the pathological changes of the placenta in association with the development of pre-eclampsia. A total of 1027 N-glyco- and 2094 phospho- sites were detected in human placental plasma membrane, of these, 5 N-glyco- and 38 phospho- proteins were differentially expressed, suggesting these proteins as candidates for biomarker of pre-eclampsia.

## Supporting Information

Table S1 **Clinical characteristics of the patients included in this study.**
(DOC)

Table S2 **1027 N-glyco- and 2094 phospho- sites detectd in this study.**
(XLS)

Table S3 **The distributions of the identified phospho- proteins and N-glyco- proteins in different chromosomes and comparison to those of gene-coding proteins in human chromosomes.**
(DOC)

Figure S1 **Mass spectra of identified N-glyco- peptides.**
(RAR)

Figure S2 **Mass spectra of identified phospho- peptides.**
(RAR)
