# Supplementary material for: Comparative N-Glycoproteomic and Phosphoproteomic Profiling of Human Placental Plasma Membrane between Normal and Preeclampsia Pregnancies with High-Resolution Mass Spectrometry
Source: PLoS One. 2013 Nov 15;8(11):e80480. doi: 10.1371/journal.pone.0080480 (PMC3829899; doi:10.1371/journal.pone.0080480)
Supplement: Table S1 — Clinical characteristics of the patients included in this study. (DOC) [file pone.0080480.s001.doc]

**Table 1. Characteristics of control and PE group**

|  | Controls (n=20 ) | Preeclampsia(n=20 ) |
| --- | --- | --- |
| Age (years) | 25.9 ± 2.7 | 26.1 ± 3.1 |
| Gestational age (weeks) | 38.3 ± 2.8 | 34.1 ± 3.2 |
| Manner of delivery | Caesarean section | |
| Systolic Blood pressure (mmHg) | 120.4 ± 6.3 | 160.7 ± 12.1* |
| Diastolic Blood pressure (mmHg) | 72.1 ± 6.8 | 97.1 ± 10.2* |
| Proteinuria (g/24h) | 0 | 3.2 ± 0.5 |
| Platelets(×10^9^/L) | 239 ± 18 | 179 ± 16 |
| ALT (U/l) | 24.1 ± 1.7 | 23.8 ± 1.5 |
| AST (U/l) | 30.8 ± 3.5 | 29.6 ± 3.2 |
| Birth Weight of infant (g) | 3024 ± 786 | 2727 ± 377 |
| Placental Weight (g) | 586 ± 193 | 495 ± 204 |

Data are presented as mean ± SEM

^*^ *P* < 0.05 compared with control
