# Supplementary material for: Comparative N-Glycoproteomic and Phosphoproteomic Profiling of Human Placental Plasma Membrane between Normal and Preeclampsia Pregnancies with High-Resolution Mass Spectrometry
Source: PLoS One. 2013 Nov 15;8(11):e80480. doi: 10.1371/journal.pone.0080480 (PMC3829899; doi:10.1371/journal.pone.0080480)
Supplement: Table S3 — The distributions of the identified phospho- proteins and N-glyco- proteins in different chromosomes and comparison to those of gene-coding proteins in human chromosomes. (DOC) [file pone.0080480.s003.doc]

The distribution of the identified phospho- proteins in different chromosomes and comparison to those of gene-coding proteins in human chromosomes.

| **Chromosome** | **Term** | **Modi. Count** | **Genome Count** |
| --- | --- | --- | --- |
| **CHROMOSOME** | 1 | 97 | 4050 |
| **CHROMOSOME** | 2 | 86 | 2805 |
| **CHROMOSOME** | 3 | 68 | 2189 |
| **CHROMOSOME** | 4 | 53 | 1724 |
| **CHROMOSOME** | 5 | 56 | 1886 |
| **CHROMOSOME** | 6 | 67 | 2776 |
| **CHROMOSOME** | 7 | 53 | 2354 |
| **CHROMOSOME** | 8 | 31 | 1615 |
| **CHROMOSOME** | 9 | 47 | 1798 |
| **CHROMOSOME** | 10 | 54 | 1635 |
| **CHROMOSOME** | 11 | 60 | 2418 |
| **CHROMOSOME** | 12 | 65 | 2025 |
| **CHROMOSOME** | 13 | 15 | 843 |
| **CHROMOSOME** | 14 | 33 | 1732 |
| **CHROMOSOME** | 15 | 32 | 1390 |
| **CHROMOSOME** | 16 | 51 | 1674 |
| **CHROMOSOME** | 17 | 54 | 2175 |
| **CHROMOSOME** | 18 | 13 | 701 |
| **CHROMOSOME** | 19 | 59 | 2347 |
| **CHROMOSOME** | 20 | 35 | 1045 |
| **CHROMOSOME** | 21 | 17 | 539 |
| **CHROMOSOME** | 22 | 31 | 1043 |
| **CHROMOSOME** | Un | 5 | 624 |
| **CHROMOSOME** | X | 58 | 2026 |
| **CHROMOSOME** | Y | 2 | 464 |

The distribution of the identified N-glyco-proteins in different chromosomes and comparison to those of gene-coding proteins in human chromosomes.

| **Chromosome** | **Term** | **Modi. Count** | **Genome Count** |
| --- | --- | --- | --- |
| **CHROMOSOME** | 1 | 62 | 4050 |
| **CHROMOSOME** | 3 | 41 | 2189 |
| **CHROMOSOME** | 4 | 27 | 1724 |
| **CHROMOSOME** | 7 | 28 | 2354 |
| **CHROMOSOME** | 11 | 34 | 2418 |
| **CHROMOSOME** | 12 | 30 | 2025 |
| **CHROMOSOME** | 13 | 9 | 843 |
| **CHROMOSOME** | 14 | 21 | 1732 |
| **CHROMOSOME** | 15 | 22 | 1390 |
| **CHROMOSOME** | 17 | 31 | 2175 |
| **CHROMOSOME** | 19 | 36 | 2347 |
| **CHROMOSOME** | 20 | 11 | 1045 |
| **CHROMOSOME** | 22 | 12 | 1043 |
